# Monitoring the evolution of vaccine-derived poliovirus in East and Southern African countries, 2010 - 2021

**DOI:** 10.11604/pamj.2024.47.31.39945

**Published:** 2024-01-25

**Authors:** Charles Byabamazima, Brine Masvikeni, Reggis Katsande, Daudi Manyanga

**Affiliations:** 1 World Health Organization, Inter-Country Support Team Office for East and Southern Africa, Belvedere, Harare, Zimbabwe,; 2 World Health Organization Regional Office for Africa, Brazzaville, Republic of the Congo

**Keywords:** Vaccine-derived poliovirus, polio eradication, classification, acute flaccid paralysis, surveillance

## Abstract

**Introduction:**

the Africa region was certified indigenous wild poliovirus-free in August 2020. Countries in East and Southern Africa have, during acute flaccid paralysis (AFP) and environmental surveillance (ES), detected equally concerning vaccine-derived polioviruses (VDPVs) that have not been systematically documented to guide programming in the sub-region. The study documents trends and salient observations of the VDPVs by country of detection, for 11 years from 2010 to 2021.

**Methods:**

we conducted secondary data analysis, a descriptive study design, by deploying field and laboratory of AFP and environmental surveillance databases of the 20 East and Southern African countries from 2010 to 2021.

**Results:**

a total of 318 VDPVs were reported over the study period. The majority were from AFP cases (58.8%) and the rest equally distributed between healthy community children and environmental surveillance sources. More polioviruses were detected after 2016 than during the period before. We observed that more boys were affected by VDPVs compared to girls. Children under 5 years were more affected than other age groups, with a mean age of 3.6 years. Delay of samples in the field seemed to increase the likelihood of not reporting VDPVs and not mounting timely public health detailed investigations and vaccination responses.

**Conclusion:**

the study provides useful evolutional trends of VDPVs for surveillance and vaccination programming. We also noted that the VDPV2s have been increasing after the 2016 tOPV to oral polio vaccine (bOPV) switch. The COVID-19 pandemic emergence in 2020, led to a decline in AFP, ES surveillance, and immunization activities. Our findings point to the need to implement enhanced tailored childhood immunization recovery strategies and to speed up the use of inactivated polio vaccine (IPV) to boost population immunity.

## Introduction

Vaccine-derived derived polioviruses (VDPVs) emerge out of the use of the oral polio vaccine (OPV) through mutation, rarely causing paralytic poliomyelitis [1]. Monitoring the evolution of VDPVs is as important for interventional actions in the polio eradication initiative as it is for wild poliovirus [2]. Low population immunity due to lack of sufficient vaccination may promote the evolution of VDPVs [3]. Oral polio vaccine has been instrumental in accelerating progress toward polio eradication ever since this initiative was declared by the World Health Assembly in 1988. Globally only two countries, Afghanistan and Pakistan are known to be still endemic with wild poliomyelitis [4]. In the WHO Africa region, Nigeria was the last endemic country for wild poliovirus [5,6]. Africa was certified indigenous wild polio-free in August 2020, which was among the key achievements of the Global polio eradication initiative [7]. The challenge remains to maintain wild polio-free certification status with high population immunity across the region while addressing the adverse effects of the COVID-19 pandemic, insecurity, geographical inaccessibility, and climatic change. To ensure that overall populations are well vaccinated and protected from wild and vaccine-derived polioviruses, the WHO introduced an immunization recovery plan [8]. Surveillance for polioviruses is an important strategy to monitor the interventions in the plan.

Polioviruses comprise 3 serotypes (PV1, PV2, and PV3) based on their capsid protein 1 (VP1). The mutated poliovirus evolves at a fairly predictable annual rate of approximately 10 nucleotide (nt) changes in its 903 nt distance of the VP1 loop [9]. The poliovirus RNA replication process makes an error estimated at 1 in every 1000 nucleotides in each round of replication in the evolution of this virus [10]. Polioviruses including wild, vaccines, and mutated variants can be excreted and transmitted alike [11].

For program intervention purposes to respond to VDPVs, the mutated polioviruses are classified as VDPV only when they surpass a VP1 sequence difference of ≥10 nucleotide changes for Sabin 1 and 3 and ≥6 nucleotide changes for Sabin 2. Polioviruse type 2 (PV2) Sabin viruses have a lower nucleotide (6) assigned threshold than PV3 and PV1. This is because, at this lower nucleotide change, PV2 viruses were observed to comparatively relate more to an outcome of paralytic cases in some countries notably Nigeria [4,11,12].

Based on clinical, immunological, and epidemiological information, for ease of programming responses, VDPVs are practically still classified into 3 groups [3,13] for the study period. The first class of circulating VDPVs (cVDPVs) are genetically linked and detected from i) two or more individuals with or without acute flaccid paralysis (AFP) from different households; or ii) one individual and at one or more environmental samples; or iii) a series of environmental samples from at least two sites; or iv) one VDPV with evidence of circulation from available genetic information about other VDPVs. The second class of VDPVs are those isolated from persons with proven immune deficiencies (iVDPV). The third class of VDPVs falls outside classes 1 and 2 and thus remains an ambiguous VDPV (aVDPV) after thorough investigations have excluded the two distinct classes, cVDPV and iVDPV. Although aVDPVs can be detected in settings of high immunization coverage, they could be important for subsequent circulation in instances of poor immunization and surveillance quality. Until now, the VDPV2 is known to be more transmissible and to circulate longer than VDPV1 and VDPV3.

Although the system of surveillance has established ways to detect, investigate, and report VDPVs, in East and Southern Africa (ESA), no systematic documentation has been done to provide an easy-to-access database on the temporal evolution of the classes of VDPV, by country. Such a database would aid countries in risk prediction based on VDPV frequency of occurrence, which would help in preparedness planning to pre-empt and interrupt further transmission. Information on VDPVs is still scattered in various laboratory and case-based databases, reports, and emails. Sometimes this information is accessed by programs from invalidated sources, and this can confuse users especially when programming some aspects of responses, including resource mobilization. Therefore, this paper is purposed to consolidate validated information from credible sources authenticated by the WHO-supported surveillance system. The documentation will provide polio eradication partners, particularly in the ESA sub-region, with reliable easy-to-access reference information for planning, monitoring, and poliovirus inventory updates for containment actions, risk assessments, and programming decisions.

## Methods

### Field and data compilation

**Study design:** we conducted a descriptive mixed qualitative and quantitative secondary analysis of data compiled from confirmed and authenticated VDPVs detected and reported from 20 countries of East and Southern Africa.

**Setting:** the data source was polio laboratory test results that yielded vaccine-derived polioviruses (VDPVs) from samples collected from the AFP and Environmental poliovirus surveillance systems in countries of East and Southern Africa, over 11 years.

**Data source:** we gathered and reviewed the available field acute flaccid paralysis and global polio laboratory network (GPLN) data from 2010 to 2021 from WHO archives and shared laboratory databases and reports, as the main source. Additionally, data from field environmental surveillance were reviewed. Individual situation, outbreak investigation, and outbreak/event response reports provided a supplement of clinical, epidemiological, and immunological information.

**Laboratory testing process:** well-validated global polio laboratory network testing methods were used to detect polioviruses [14]. Before May 2016 (tOPV to bOPV switch) polioviruses that were Sabin-like (SL) were reported as final results. After 2016, all poliovirus type 2 (PV2) from the Africa polio laboratory network were referred to a sequence testing laboratory, mostly the National Institute for Communicable Diseases (NICD), but sometimes to the Global Specialised Polio Laboratory at the Centres for Diseases Control (CDC) in the USA, and more recently the National Institute of Biological Standards (NIBSC), U.K [15]. All polioviruses of the 3 serotypes that were discordant (Sabin-like on ITD tests and non-Sabin-like or NSL on VDPV screening tests) before 2016 were outrightly referred to the sequencing laboratories for confirmatory testing.

By genomic sequencing, polioviruses were grouped as VDPV type 1 or, type 3 if the nucleotide sequence change was > 1% for serotypes 1 and 3 in the ∼900-nucleotide VP1 sequence encoding capsid protein VP1 and >0.6% for serotype 2 compared to the parent OPV strain [13]. The VDPVs were classified following the operational WHO standard guidelines published in August 2016, additionally putting into consideration the clinical, epidemiological, and immunological information after appropriate investigation [11,16]. The classified VDPVs detected in countries of East and Southern Africa are presented in this paper by serotype and year of onset or detection depending on specimen source (AFP/human and or environmental sample).

## Results

A total of 318 VDPV with representation of the three serotypes were detected and documented from 9 countries of East and Southern Africa namely Eritrea, Ethiopia, Kenya, Madagascar, Mozambique, South Africa, South Sudan, Uganda, and Zambia for the period of 1 January 2010 to 31 December 2021. The majority of these VDPVs (58.8%) were isolated from the AFP cases, 22.3% from healthy community contacts, and a further 22.3% from environmental surveillance (Table 1). We observed a 39.9% difference (95% CI range 32.7% - 46.5%) between VDPVs reported from AFP cases compared to those from environmental samples, with a Chi-squared value of 106.383 and a p-value < 0.0001 demonstrating a statistically significant difference.

**Table 1:** distribution of vaccine-derived poliovirus types by sample source and country of detection, 2010 - 2021, in East and Southern African countries

Reporting Country	cVDPV 1	cVDPV 2	cVDPV 3	aVDPV 1	aVDPV 2	aVDPV 3	iVDPV 3
Eritrea	0	2021:1 (A)	0	0	0	0	0
Ethiopia	0	2019: 14 (A), 2020: 36 (A), 2021: 10 (A), 2019: 9 (C) 2020: 7 (C) 2019: 3 (E), 2020: 4 (E)	2010:5 (A)	0	2012:1(A),2014:1 (A), 2015:1(A),2020: 3 (A), 2021:1(A),2020: 1 (E), 2021: 3 (E)	2010:1(A)	0
Kenya	0	2012:3 (A), 2020:1 (C), 2021:2 (C), 2018:1 (E), 2020:1 (E), 2021:1 (E)	0	0	2021:1 (A), 2015:1 (E), 2018:3 (E), 2021: 3 (E)	0	0
Madagascar	2014: 1 (A), 2015:10 (A), 2020: 2 (A), 2021:13 (A), 2021:25 (C), 2021:31 (E),	0	0	2015:1(A), 2021:1(C)	0	0	0
Mozambique	2011:2 (A), 2020: 1 (A),	2018:1 (A), 2018:2 (C), 2021: 2 (A),	0	2019:1 (A) 2020:1 (A)	2016:1(A),2018:1(A), 2019: 1 (A),	0	0
South Africa	0	0	0	0	0	0	2011: 1 (A), 2017: 1 (A) 2018: 1 (A), 2021: 1 (A)
South Sudan	0	2014: 2 (A), 2020: 50 (A), 2021: 9 (A), 2020: 19 (C), 2021: 5 (C) 2020: 6 (E)	0	0	2012: 1 (A), 2015: 1 (A)	0	0
Uganda	0	2021: 2 (E)	0	0	2014: 2 (A)	0	0
Zambia	0	2019: 2 (A), 2019: 2 (C)	0	0	0	0	0
**Total**	**85**	**193**	**5**	**4**	**26**	**1**	**4**

* A = AFP cases **E= Environmental surveillance, ***C=Community health contacts; vaccine-derived polio viruses circulating (cVDPVs); aVDPVs: ambiguous vaccine-derived polio viruses; iVDPVs: immune deficiencies vaccine-derived polio viruses

In terms of geographical distribution during the study period, the majority of VDPVs were reported from Ethiopia (31.4%) for over 7 years out of 11 years, South Sudan (29.2%) reported over 5 years, and Madagascar (26.4%) over 4 years period (Table 1). In addition, one iVDPV3 was reported from South Africa, while only Ethiopia reported aVDPV3 and cVDPV3 during the study period. The majority of cVDPV1 (96.5%) were reported from Madagascar and spread over a total duration of 4 years. Mozambique reported cVDPV1, cVDPV2, aVDPV1and aVDPV2 in 6 years, the last four years of the study being 2018, 2019, 2020 and 2021. No VDPVs were reported from the environmental samples in the country over the last 4 years of the study.

Regarding the dominating serotype, we observed that during the study period, the majority (60.7%) of the reported VDPVs were cVDPV2, followed by cVDPV1 (26.1%) while iVDPV1 and iVDPV2 were not reported at all (Table 2). Surprisingly, 85.8% of all isolated VDPVs were reported in the three years of the study (01 January 2018 to 31 December 2021) compared to 14.2% in the remaining 9 years (2010 to 2018). The distribution of VDPV by year indicates that 41.5% of VDPV were reported in 2020 and none in 2013 (Table 2).

**Table 2:** evolution and classification of vaccine-derived polioviruses 2010 - 2021, in East and Southern African countries

VDPV Type	2010	2011	2012	2013	2014	2015	2016	2017	2018	2010-2018 n (%)	2019	2020	2021	2019-2021 n (%)	Total n (%)
cVDPV1	0	2	0	0	1	10	0	0	0	13 (28.9)	0	3	69	72 (26.4)	**85 (26.7)**
cVDPV2	0	0	3	0	2	0	0	0	4	9 (20.0)	30	124	30	184 (67.4)	**193 (60,7)**
cVDPV3	5	0	0	0	0	0	0	0	0	5 (11.1)	0	0	0	0 (0.0)	5 (1.6)
**Subtotal cVDPV**	**5**	**2**	**3**	**0**	**3**	**10**	**0**	**0**	**4**	27 (60.0)	**30**	**127**	**99**	256 (93.8)	283 (89)
aVDPV1	0	0	0	0	0	1	0	0	0	1 (2.2)	1	1	1	3 (1.1)	4 (1.3)
aVDPV2	0	0	2	0	3	3	1	0	4	13 (28.9)	1	4	8	13 (4.8)	26 (8.2)
aVDPV3	1	0	0	0	0	0	0	0	0	1 (2.2)	0	0	0	0 (0.0)	1 (0.3)
**Subtotal aVDPV**	**1**	**0**	**2**	**0**	**3**	**4**	**1**	**0**	**4**	15 (33.3)	**2**	**5**	**9**	16 (5.9)	31 (9.7)
iVDPV1	0	0	0	0	0	0	0	0	0	0 (0.0)	0	0	0	0 (0.0)	0 (0.0)
iVDPV2	0	0	0	0	0	0	0	0	0	0 (0.0)	0	0	0	0 (0.0)	0 (0.0)
iVDPV3	0	1	0	0	0	0	0	1	1	3 (6.7)	0	0	1	1 (0.4)	4 (1.3)
**Subtotal iVDPV**	**0**	**1**	**0**	**0**	**0**	**0**	**0**	**1**	**1**	3 (6.7)	**0**	**0**	**1**	1 (0.4)	4 (1.3)
**Total (all VDPVs)**	**6**	**3**	**5**	**0**	**6**	**14**	**1**	**1**	**9**	45 (100)	**32**	**132**	**109**	273(100)	318 (100)

Vaccine-derived polio viruses (VDPVs); vaccine-derived polio viruses circulating (cVDPVs); aVDPVs: ambiguous vaccine-derived polio viruses; iVDPVs: immune deficiencies vaccine-derived polio viruses

In the analysis of secondary data of the reported AFP cases from where VDPVs were isolated, we found that the age range among the cases was 0 months to 22 years, the mean age being 3.6 years (range 3.09 - 4.09 yrs.), while the median was 2.5 years. Without age, records were 2.7% of the total number. Most of the AFP cases with VDPV were children aged 0-59 months (78.6%) followed by 5- 9 years (15.5%) and those over 9 years (4.8%) (Table 3). The gender of the majority of the reported AFP cases was male (54.5%) while female was 42.7%. We observed an 11.8% difference (95% CI ranges 2.78% to 25.66%) between males and females, with a chi-squared value of 2.484 and p-value of 0.1115, indicating no statistically significant difference.

**Table 3:** characteristics (parameters) of the reported vaccines-derived poliovirus cases, 2010-2021 in East and Southern African countries

Parameters	2010	2011	2012	2013	2014	2015	2016	2017	2018	2019	2020	2021	Total
**Age group**													
Missing	0	0	0	0	0	0	0	0	0	0	2	0	2
0 - 11 months	0	0	0	1	0	2	0	1	0	1	9	0	14
1 - 4 yrs	8	3	3	13	4	7	0	0	0	15	60	20	133
5 - 9 yrs	2	0	1	2	0	1	1	0	1	1	14	6	29
10 - 14 yrs	0	0	0	0	0	1	0	0	0	0	4	0	5
15+ yrs	0	0	0	4	0	0	0	0	0	0	0	0	4
**OPV doses (0-59 months)**													
Zero doses	0	3	0	8	1	9	0	0	1	3	14	4	43
<3 Doses	6	0	3	2	0	0	0	1	0	7	16	7	42
3 Doses +	2	0	0	4	3	0	1	0	0	6	39	9	64
**Hospital admission status**													
Missing	2	1	2	6	3	0	0	0	0	7	23	3	47
Admitted	2	1	0	5	1	4	0	1	0	6	24	12	56
Not admitted	6	1	2	9	0	7	1	0	1	4	42	11	84

Regarding vaccination status of the 0-59 months age group, 43.0% received 3 or more OPV doses, 28.1 received less than 3 OPV doses and 28.9% had no history of being vaccinated. Most (44.9%) of the reported AFP cases where VDPV was isolated, were not admitted; only 30.0% reported to have been admitted, while for 25.1% of the admissions, the status information was missing. Although there is a 14.9% (95% CI ranges from -1.48% for 29.86%) variance and a p-value of 0.0756 for those who were not admitted compared to those admitted, no difference of statistical significance was observed.

The presentation among reported AFP cases was with the following clinical pictures; 83.4% fever, 38.5% asymmetrical paralysis, 36.9% symmetrical paralysis, and 74.3% progression of paralysis reported to be within 3 days (Table 4). Furthermore, we noticed that the majority (59.9%) of AFP cases from whom VDPV was isolated had their stool samples delivered within 7 days in the polio laboratories from the day of collection of the second stool samples (Table 4). We observed a 31.6% (95 CI ranges from 15.45% to 45.05%) difference between samples delivered within 7 days compared to after 7 days, with a Chi-squared value of 14.283 and a p-value of 0.0002 which was statistically significant. It was noted that 7% of laboratory results of the AFP cases were processed in more than 30 days of receiving the samples.

**Table 4:** clinical presentations and timelines for shipment and processing stool samples of the reported vaccines-derived poliovirus cases, 2010-2021 in East and Southern African countries

Parameters	2010	2011	2012	2013	2014	2015	2016	2017	2018	2019	2020	2021	Total
**Fever at onset**													
Missing	0	0	0	6	1	0	0	0	0	2	5	5	19
Yes	10	3	4	14	3	11	1	1	1	14	74	20	156
No	0	0	0	0	0	0	0	0	0	1	10	1	12
**Presentation of paralysis**													
Missing	0	0	0	7	1	0	0	0	0	5	26	7	46
Asymmetrical	4	2	3	8	0	2	1	0	1	9	32	10	72
Symmetrical	6	1	1	5	3	9	0	1	0	3	31	9	69
**Progression of paralysis**													
Missing/Unknown	0	0	0	9	1	0	0	0	0	4	11	8	33
Yes	9	3	4	11	0	9	1	1	1	12	71	17	139
No	1	0	0	0	3	2	0	0	0	1	7	1	15
**Duration for the lab to receive samples after collection of second stool**													
Missing	1	0	0	1	0	0	0	1	0	0	17	2	22
Within 3 Days	9	1	2	8	1	3	0	0	0	16	38	16	94
4-7 Days	0	0	1	9	1	3	1	0	0	1	1	1	18
Over 7 Days	0	2	1	2	2	5	0	0	1	0	33	7	53
**Days for results were out after the specimen receipts**													
Missing	6	0	0	4	1	1	0	1	0	1	22	2	38
Within 30 Days	4	3	4	16	2	10	1	0	1	15	63	17	136
Over 30 Days	0	0	0	0	1	0	0	0	0	1	4	7	13

## Discussion

Between 2010 and 2021, all three VDPV serotypes were detected in the ESA sub-region and cVDPV2 was the most predominant which has also been observed in other WHO Africa sub-regions [4,11,17]. This is consistent with the expected waning natural immunity against Poliovirus type 2 since Wild poliovirus (WPV)2 was last detected globally in 1999 and the global switch of top to bOPV -where serotype 2 was dropped from the oral polio vaccine and replaced by the component available in the inactivated polio vaccine (IPV) occurred in 2016 [18]. The disruption of immunization services and mass vaccination to prevent (or respond to) polio outbreaks in 2020, due to both the COVID-19 pandemic restrictions and related repurposing of the health workforce in response, may have contributed to the increased number of VDPVs in the year. We also realized that though environmental surveillance is implemented in most of the reporting countries (except Eritrea) the majority of VDPVs were detected from AFP cases. This calls for the need to revamp or review both AFP and ES strategies to see the contribution and impact of each with a focus on the environmental surveillance in some countries that never reported VDPVs for the 11-year study period. On the other hand, in countries with no reported VDPVs for the period of this study, investigation of the profile of population immunity could best explain the possible root cause for the absence of VDPV evolution, particularly where AFP surveillance standards have consistently met the WHO requirements. The domination of cVDPV1 in Madagascar, recurring 6 times over the 11 years calls for additional in-depth exploratory research to identify the specific determinants for the population variability of the population immunity following OPV use.

Capacity building for health workers including strengthening laboratory capacities and infrastructure in countries in most ESA sub-region countries may ensure all iVDPV are also detected and reported as was the case in South Africa. The observation of cases not fitting the clinical AFP standard case definition (unexpected cases with progression of paralysis in more than 3 days and/or without fever), suggests that the inadequately skilled personnel may miss candidate clinical AFP cases with VDPVs. If countries in the sub-region continue using OPV, the risk of detecting the VDPV remains and in some situations may increase particularly for PV2 that is not in the routine OPV vaccine composition. Fast-tracking the full introduction of IPV into countries´ immunization programs may have added value to minimizing the risk of paralytic polio due to the VDPVs. We also realized, that in some countries where there are recurrent reports of VDPVs, there are some considerable well-known long-standing population immunity gaps. We suggest, as part of the outbreak response, that simultaneous efforts be made to strengthen routine immunization and surveillance systems during and after the periodic outbreak responses to VDPVs. The observation that most VDPV were isolated from stool samples delivered to the laboratory within 7 days of collection of the second stool, points to a possibility of considerable loss of polioviruses in samples that delay 7 days suggesting the need to invest in ways to promote faster shipment and reliable cold storage for samples that cannot be timely delivered for inevitable reasons.

**Limitations and how we managed them not to derail findings:** data generated before 2010 was unreliable and thus could not be included because of inconsistencies between different databases. The classification of the VDPV from AFP cases and those detected from human contacts and environmental surveillance viruses is influenced by the level of sensitivity of surveillance, availability of advanced immunological laboratory diagnostic facilities, and good clinical acumen to detect primary immunodeficiencies to facilitate VDPV classification and decisions on the type and scale of vaccinations response. At the country level, it was not possible to explain why Madagascar continued reporting cVDPV1 but not the other VDPV serotypes, and yet the country is still using bOPV for routine vaccination as for other ESA countries. From this study, it was also not possible to understand why the yield of viruses from environmental surveillance in Mozambique (and Zambia) was poor over the years of observation and therefore call for tailored research to address the underlying factors that could include knowledge and operational gaps.

## Conclusion

The pattern of evolution of VDPV within ESA countries has been presented with the VDPV trajectories in some countries such as Ethiopia, South Sudan, Madagascar, Mozambique, and Kenya over the study period. The contribution of the impact of the COVID-19 pandemic to increased VDPV reporting has been observed. The effects of delays of stool samples in VDPV yield are apparent and the need for expediting immunization services resumption to the ESA countries to boost population immunity is suggested by the data. The rare detection of iVDPVs requires deliberate investment in capacity enhancement at the clinical and immuno-laboratory investigational levels. This study also points to an urgent need to improve stool shipment to the laboratories as for proper field storage conditions.

### What is known about this topic

- *Vaccine-derived polioviruses (VDPV) can emerge and or be imported into a geographical area where there are population immunity gaps/declined immunity of the specific poliovirus strain;*
- *Vaccine-derived polioviruses type-2 (VDPV2) were envisaged to emerge after the 2016 switch from trivalent oral polio vaccines (tOPV) to bivalent oral polio vaccine (bOPV);*
- *Countries using oral polio vaccine (OPV) in both routine and mass vaccination in the form of campaigns are at risk of VDPV emergency, as long as they have significant gaps in population immunity- a situation that was aggravated by COVID-19 pandemic disruption of routine vaccination services in most ESA countries due to decline of OPV vaccination coverage*.

### What this study adds

- *The study identifies present VDPV epidemiological and risk trajectories in ESA (and potentially other) countries and avails data for programmatic decision-making based on the magnitude, frequency of occurrence, and types of the VDPV - for improved prediction and preparedness planning;*
- *The documentation provided health workers in the sub-region an opportunity for motivation to strengthen surveillance and to understand better the essence and application of exhaustive VDPV investigations for classification in the context of informed programming, while also identifying critical gaps for further study - in countries where it could not be possible to explain the patterns and periodicity of certain types of VDPVs;*
- *It reinforces to the countries and immunization partners the urgency to expedite the introduction of a complete vaccination schedule for inactivated polio vaccine (IPV) and to assure increased vaccination coverage for applicable polio vaccines in all countries in and beyond ESA*.
